# Glycine as a Metabolic Regulator of Reproductive Function in Livestock: From Gametes to Early Embryos

**DOI:** 10.3390/ani16131967

**Published:** 2026-06-25

**Authors:** Yuxin Teng, Chenjun Wang, Yingjie Wu, Chang Yan, Yinghe Qin

**Affiliations:** State Key Laboratory of Animal Nutrition and Feeding, College of Animal Science and Technology, China Agricultural University, No. 2 Yuanmingyuan West Road, Haidian District, Beijing 100193, China; tengyuxin@cau.edu.cn (Y.T.); wangchenjun@cau.edu.cn (C.W.); wuyingjie@cau.edu.cn (Y.W.); yanchang@cau.edu.cn (C.Y.)

**Keywords:** glycine, animal reproduction, oocyte maturation, embryonic development, redox homeostasis, one-carbon metabolism, nutritional strategy

## Abstract

Reproductive inefficiency associated with impaired oocyte competence and embryonic loss remains a major limitation in livestock production. Although glycine is classified as a non-essential amino acid, its endogenous synthesis is often insufficient to meet increased metabolic demands during gestation and early embryonic development. This suggests that glycine has a conditionally essential role in reproductive physiology. However, the mechanisms through which glycine integrates metabolic and signaling processes to regulate reproductive outcomes are not fully understood. This review summarizes the recent advances in understanding glycine’s role in animal reproduction, emphasizing its function as a metabolic regulator rather than merely a structural component. Glycine contributes to reproductive processes by maintaining redox homeostasis, supporting mitochondrial function and stabilizing cellular environments as part of its osmolyte function during critical developmental stages. Additionally, glycine participates in one-carbon metabolism, influencing nucleotide synthesis and epigenetic regulation. Furthermore, emerging evidence suggests that glycine may modulate key signaling pathways, including the AMP-activated protein kinase (AMPK)-mechanistic target of rapamycin complex 1 (mTORC1) pathway. Consistent with these mechanistic roles, glycine supplementation has been associated with improvements in oocyte maturation and embryonic development, particularly in vitro. These findings highlight the potential of glycine as a dietary or culture medium supplement to enhance reproductive performance in livestock. However, most current evidence is derived from in vitro systems, and the translation of these findings into livestock production strategies requires validation through well-designed in vivo studies.

## Simple Summary

Glycine is a simple amino acid that performs many essential jobs in the animal body. Although its roles in maintaining general health are well known, its importance for animal fertility is often overlooked. Poor oocyte quality, reduced sperm function, and early embryonic death are major causes of reproductive failure in pigs, cattle, sheep, and poultry, leading to substantial economic losses in livestock farming. This review summarizes current evidence on how glycine may influence reproductive cells and early embryos. Available studies suggest that glycine can help maintain redox balance, mitochondrial function, osmotic stability, and metabolic signaling, especially in in vitro culture or preservation systems. However, much of the current evidence comes from laboratory models, and responses may differ among species, doses, and experimental conditions. Further in vivo studies are needed before glycine-based nutritional or culture strategies can be reliably applied to improve livestock reproductive performance.

## 1. Introduction

As the global population continues to grow, increasing demands will be placed on livestock production systems to ensure a stable supply of high-quality animal-derived food. Reproductive efficiency is a key determinant of productivity in animal agriculture. However, embryonic loss and suboptimal reproductive performance remain major constraints. In beef cattle, embryonic loss can exceed 47% by day 30 of gestation, and in sows, total embryonic and fetal mortality can reach over 50%, with the majority of losses occurring during the early stages of pregnancy [1,2,3]. These inefficiencies not only reduce production profitability but also limit the sustainability of modern animal production systems.

Successful animal reproduction is a tightly regulated biological process encompassing oocyte maturation, fertilization, early embryonic development, implantation, placental formation, fetal growth, and parturition [4]. Disturbances at any of these stages, especially those occurring during early embryogenesis, can markedly impair reproductive efficiency. Reproductive success is affected by numerous factors, including maternal nutritional status, endocrine profile, uterine environment, and genetic regulation [5,6,7]. Among these factors, nutritional regulation is considered one of the most pivotal, since it directly governs metabolic homeostasis, redox balance, and diverse cellular signaling pathways, consequently impacting oocyte quality and embryonic developmental potential [8,9,10,11,12].

Among the various nutritional inputs, functional amino acids have emerged not merely as building blocks but as crucial signaling entities that dictate gamete competence and uterine receptivity. However, compelling evidence in rapidly growing neonates and gestating females indicates that the endogenous synthesis of glycine frequently fails to meet metabolic demands, rendering it a conditionally essential amino acid in these physiological states, such as rapid growth, gestation, and elevated reproductive activity [13,14]. Although mild glycine deficiency is not acutely life-threatening, chronic insufficiency has been linked to compromised immune function, suboptimal growth, and widespread metabolic disorders [15,16,17]. In these scenarios, glycine availability may act as a metabolic bottleneck, especially in highly proliferative and metabolically active tissues, including the gonads and early embryonic tissues [18,19].

Recently, accumulating evidence has demonstrated that in vitro supplementation with exogenous glycine promotes oocyte maturation, improves embryonic developmental competence, and maintains mitochondrial function [20,21,22]. Furthermore, glycine participates in one-carbon metabolism [23], thus exerting regulatory effects on epigenetic modifications and gene expression during early embryonic development [24,25].

Functional amino acids are relevant in this context because they serve as metabolic substrates and signaling-related molecules. Glycine is traditionally classified as non-essential, but endogenous synthesis may become limiting during reproductive states characterized by rapid cell proliferation and high metabolic flux, including folliculogenesis, spermatogenesis, and placentation. Clarifying how glycine supports these processes may help refine dietary strategies for breeding animals and improve culture or preservation media used in assisted reproductive technologies, provided that in vitro findings are validated under physiologically relevant conditions.

In this review, we summarize recent progress on the roles of glycine in animal reproduction, with a special emphasis on its effects on oocyte quality and embryonic development. We further elaborate on the underlying molecular mechanisms, including maintenance of redox homeostasis, improvement of mitochondrial function, regulation of one-carbon metabolism, and modulation of critical signaling pathways. This review aims to provide a balanced theoretical basis for evaluating the potential application of glycine in livestock reproduction, while highlighting current evidence gaps and the need for further in vivo validation.

## 2. Methods

This review was conducted as a narrative synthesis of peer-reviewed literature examining the roles of glycine metabolism in animal reproduction. Literature was identified through searches of PubMed, Web of Science, and Scopus databases mainly covering the period from 2005 to 2026, with particular emphasis on studies published within the past five years. Earlier seminal studies were also incorporated where contextually relevant. Search terms included combinations of “glycine” with “livestock”, “reproduction”, “oocyte”, “sperm”, “embryo”, “fetal development”, “one-carbon metabolism”, “glutathione”, “oxidative stress”, “mitochondrial function”, “AMPK”, “mTOR”, and “epigenetics”. Species-related terms including “pig”, “porcine”, “cattle”, “bovine”, “sheep”, “poultry”, and “chicken” were also incorporated. Additionally, reference lists of retrieved articles were manually screened to identify additional relevant publications. Due to the limited availability of in vivo evidence in certain areas, both in vivo and in vitro studies were discussed, and mechanistic studies from non-livestock models were considered when they provided relevant biological context. The evidence was synthesized according to reproductive stage, biological mechanism, and physiological relevance, with particular attention to distinguishing direct livestock evidence from indirect or hypothesis-generating findings.

## 3. Physiological Functions of Glycine

Glycine is the simplest proteinogenic amino acid, with a hydrogen atom as its side chain [26]. This unique structural feature renders glycine the only achiral amino acid, with no D or L-isomers. Glycine has a sweet taste, and its name is derived from the Greek word “glykys,” meaning sweet [27]. Glycine accounts for 11.5% of the total amino acids in the human body, and 20% of total amino acid nitrogen in human proteins [28]. In several mammals and humans, a large proportion of whole-body glycine flux is channeled into protein synthesis [29].

Collagen and elastin represent the most abundant extracellular matrix proteins in the body, with glycine as their major constituent amino acid. Notably, glycine accounts for approximately one-third of all amino acid residues in collagen [30]. Structurally, collagen comprises three α-chains that intertwine to form its signature triple-helical structure. Within each α-chain, glycine residues are highly conserved and located at every third position. This repetitive motif is critical for maintaining the tight packing and stability of the triple helix. Owing to its minimal steric hindrance, glycine is uniquely capable of fitting into the interior core of the triple helix, thus playing an indispensable role in stabilizing the entire molecular structure of collagen [31]. Given these structural and functional features, an adequate glycine supply is particularly critical during physiological states characterized by rapid tissue turnover, including growth and gestation, where it sustains tissue development, remodeling, and repair [32,33].

In addition to its indispensable structural role in collagen, glycine functions as a versatile signaling molecule both centrally and peripherally. As an important inhibitory neurotransmitter in the central nervous system, glycine regulates feeding behavior, locomotor activity, and systemic homeostasis [34]. Beyond its roles in the nervous system, glycine also serves as a signaling molecule in peripheral tissues, where it modulates a wide spectrum of cellular processes [35,36]. Glycine can regulate intracellular Ca^2+^ concentration by activating ligand-gated chloride channels on immune cells, thereby modulating superoxide production and cytokine secretion and contributing to immune regulation and anti-inflammatory responses [37,38].

Glycine is also an essential precursor for glutathione (GSH) synthesis [39], the primary intracellular antioxidant that plays a vital role in maintaining redox balance and eliminating reactive oxygen species (ROS) [40]. Insufficient glycine availability can limit GSH synthesis [39], resulting in increased oxidative stress and impaired reproductive cell function [41].

Furthermore, glycine serves as a direct precursor in purine nucleotide synthesis, supplying the requisite carbon and nitrogen for DNA and RNA production to sustain rapid cell proliferation in early embryos [23]. Glycine is also involved in heme biosynthesis and creatine production, which contribute to oxygen transport and cellular energy metabolism [42,43].

Together, these functions position glycine as an important metabolic and signaling molecule linking structural integrity, neuronal signaling, antioxidant capacity, nucleotide synthesis, and energy metabolism.

## 4. Synthesis and Metabolism of Glycine

**Figure 1:**
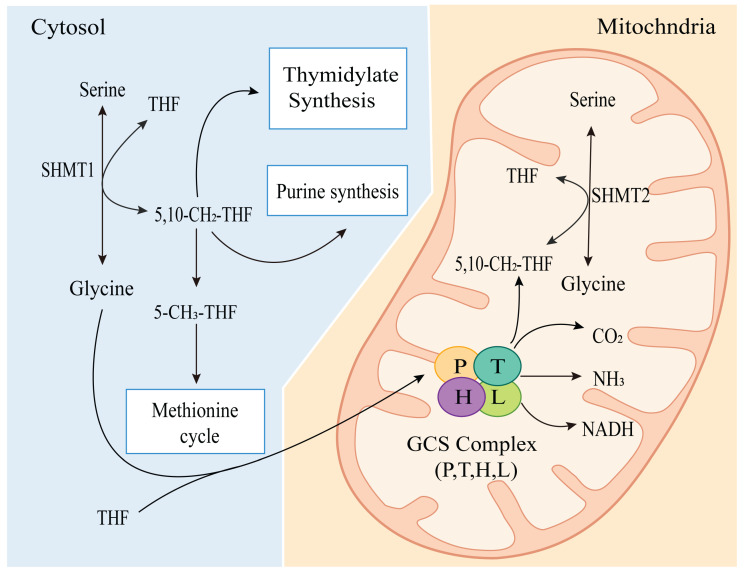
Overview of serine–glycine interconversion and one-carbon metabolism pathways. In the cytosol, serine and tetrahydrofolate (THF) are converted by serine hydroxymethyltransferase 1 (SHMT1) to glycine and 5,10-methylenetetrahydrofolate (5,10-CH_2_-THF). 5,10-CH_2_-THF subsequently serves as a key branch-point metabolite that participates in thymidylate (dTMP) and purine biosynthesis or is converted into 5-methyltetrahydrofolate (5-CH_3_-THF) to enter the methionine cycle. In mitochondria, serine is converted to glycine by serine hydroxymethyltransferase 2 (SHMT2). Both the mitochondrial and cytosolic forms of glycine are broken down by the glycine cleavage system (GCS), which is composed of P-, T-, H- and L-proteins. This process yields 5,10-CH_2_-THF, CO_2_, NH_3_ and nicotinamide adenine dinucleotide (reduced form) (NADH). Downstream one-carbon metabolism pathways are highlighted in blue boxes: thymidylate synthesis, methionine cycle, and purine synthesis.

The diverse physiological functions described above impose distinct and tissue-specific demands on glycine availability. Therefore, a tightly regulated metabolic network is required to balance de novo synthesis, dietary intake, and catabolism [23,44,45]. These metabolic processes determine the availability and utilization of glycine, and also influence key biological functions such as nucleotide biosynthesis, methylation reactions, and cellular redox homeostasis by generating and transferring one-carbon units [46] (Figure 1). Accordingly, a systematic understanding of glycine synthesis and degradation pathways is essential for elucidating its roles in cellular regulation and animal reproduction.

### 4.1. Endogenous Synthesis of Glycine

Glycine can be synthesized in the body from various precursors, including serine, threonine, choline, sarcosine (N-methylglycine), glyoxylate and L-carnitine [16]. Previous nutritional and isotope-tracing studies have demonstrated that glycine can be synthesized in pigs, humans, and other mammals, with the serine-derived pathway being the predominant source [13,47]. Subsequent biochemical studies in rats have confirmed this, further demonstrating that glycine is synthesized primarily via serine hydroxymethyltransferase (SHMT), choline-derived sarcosine metabolism and threonine dehydrogenase pathways [48,49].

Under typical dietary conditions, serine hydroxymethyltransferase activity is estimated to account for the majority (>85%) of endogenous glycine flux [50]. In mammals, glycine and serine can be interconverted via SHMT, a pyridoxal phosphate-dependent enzyme that exists in two forms: cytosolic SHMT1 (cSHMT) and mitochondrial SHMT2 (mSHMT), which are encoded by different genes [51]. In mitochondria, SHMT2 serves as the primary enzyme for glycine synthesis and catalyzes the formation of 5,10-methylene-tetrahydrofolate (5,10-CH_2_-THF), which provides essential one-carbon units for cellular metabolism [52,53,54]. 5,10-CH_2_-THF subsequently acts as a pivotal branch-point metabolite, directing metabolism towards either thymidylate (dTMP) and purine biosynthesis, or conversion into 5-methyl-THF to support the methionine cycle [55]. Therefore, the serine–glycine interconversion pathway is a key step in amino acid metabolism and a major entry point into the one-carbon metabolic network [56]. In juvenile rats, SHMT activity is higher and is accompanied by increased isotopic flux, suggesting that this pathway is particularly active during growth and development [49]. Additionally, SHMT expression exhibits tissue-, species-, and developmental stage-specific differences [57,58].

Although choline does not directly contribute carbon skeletons to glycine synthesis, it indirectly regulates glycine metabolism by influencing one-carbon metabolism and the availability of methyl donors [59,60]. Choline is oxidized to betaine, which promotes the remethylation of homocysteine to methionine, thereby supporting the generation of S-adenosylmethionine (SAM), the principal methyl donor in cells [59]. Under the catalysis of glycine N-methyltransferase (GNMT), glycine accepts a methyl group from SAM to form sarcosine [61]. Sarcosine is then demethylated by sarcosine dehydrogenase in a tetrahydrofolate (THF)-dependent manner, regenerating glycine and yielding 5,10-CH_2_-THF [62]. This cycle tightly links glycine metabolism with cellular methyl donor pathways, thereby playing an important role in regulating DNA methylation and nucleotide synthesis [45].

Threonine can also be converted to glycine via the threonine dehydrogenase (TDH) pathway, which primarily occurs in the mitochondria [63]. In this process, threonine is first oxidized to 2-amino-3-ketobutyrate, which is then cleaved into glycine and acetyl-CoA [64], thereby providing substrates for one-carbon and energy metabolism. TDH is regarded as a key rate-limiting enzyme in threonine catabolism in mammals including pigs and rodents, responsible for approximately 80% of threonine degradation in vivo [65]. Additionally, threonine can be converted to glycine via the threonine aldolase (TA) pathway. However, due to the low activity of TA in mammals [66], this pathway contributes minimally to overall glycine production.

### 4.2. Glycine Catabolism and the One-Carbon Metabolism

Glycine catabolism occurs mainly via two pathways: the reversible interconversion between glycine and serine mediated by SHMT, and the mitochondrial glycine cleavage system (GCS) [67]. Both pathways employ THF as a one-carbon carrier, thus tightly linking glycine metabolism to the generation of one-carbon units [68].

During SHMT-mediated reactions, glycine can be converted to serine while generating 5,10-CH_2_-THF, which is a key one-carbon donor for nucleotide biosynthesis and methylation reactions [69]. During spermatogenesis, oocyte maturation, and early embryonic development, the rapid replication of DNA and cell division create a high demand for nucleotides [46,70]. Therefore, the efficiency of the serine–glycine–one-carbon metabolic pathway may directly influence the proliferative capacity and genomic stability of reproductive cells. Furthermore, 5,10-CH_2_-THF produced via glycine metabolism is an important contributor to folate metabolism [71]. Folate plays a critical and indispensable role during embryonic development. Maternal folate deficiency can impair one-carbon metabolism, resulting in complications including intrauterine growth restriction (IUGR) and aberrant DNA methylation [72,73]. Taken together, these findings indicate that the glycine–serine interconversion indirectly favors reproductive outcomes through its participation in one-carbon metabolism.

The glycine cleavage system (GCS) is another major pathway for glycine degradation. Located in mitochondria, the glycine cleavage system (GCS) is a multienzyme complex comprising glycine decarboxylase (GLDC, P-protein), aminomethyltransferase (AMT, T-protein), GCS protein H (GCSH), and dihydrolipoamide dehydrogenase (DLD, L-protein). GCS catalyzes the degradation of glycine to produce CO_2_, transfers one-carbon units to THF to form 5,10-CH_2_-THF, while releasing NH_3_ and generating nicotinamide adenine dinucleotide (reduced form) (NADH) [74]. One-carbon units derived from GCS can be utilized for serine and methionine synthesis and exhibit tissue-specific distribution patterns [75]. Furthermore, modulating key components of the GCS, such as the lipoylation status of the H protein, can significantly enhance its metabolic activity [76], thereby increasing the flux of glycine into one-carbon metabolism. Notably, abnormalities in GCS-related genes are closely associated with embryonic developmental defects (such as neural tube defects) [45,77], further underscoring the crucial role of glycine catabolism and its one-carbon metabolic function in embryonic development.

## 5. Effects of Glycine on Gamete, Embryo, and Fetal Development

### 5.1. Effects of Glycine on Oocyte Maturation and Developmental Competence

In mammalian species such as cattle, pigs, and mice, oocyte maturation involves a highly orchestrated sequence of nuclear and cytoplasmic remodeling, which is fundamental to achieving embryonic developmental competence [78]. Studies on bovine and porcine assisted reproduction have demonstrated that oocytes cultured in vitro are significantly more vulnerable to environmental stressors than their in vivo counterparts. These stressors, including oxidative stress, osmotic imbalance, and metabolic shifts, collectively compromise oocyte quality and limit developmental potential [79,80]. Specifically, the oxidative stress mainly triggered by excessive accumulation of ROS has been identified as a primary driver of mitochondrial dysfunction, DNA fragmentation, and the induction of apoptosis in these mammalian models [81,82].

Analysis of porcine follicular fluid showed that glycine was one of the predominant amino acids, with its concentration decreasing in a stage-dependent manner from 562.01 ± 27.56 μM in small follicles to 336.46 ± 8.82 μM in large follicles [83,84]. This pattern indicates a potential association between glycine availability and follicular development. In porcine IVM systems, glycine supplementation has been reported to improve oocyte maturation and subsequent embryonic development. For instance, glycine has been reported to enhance cleavage and blastocyst formation rates in porcine oocytes [21]. These improvements are generally associated with enhanced intracellular redox balance, improved mitochondrial function [85], and cellular homeostasis under in vitro culture conditions [20]. Furthermore, glycine functions as a potent organic osmolyte, facilitating cell volume regulation and adaptive responses to osmotic stress [86,87,88]. Given that osmotic pressure fluctuations disrupt cytoskeletal organization, mitochondrial distribution, and meiotic spindle integrity in oocytes, glycine-mediated stabilization of intracellular osmotic balance may further safeguard oocyte structural integrity and developmental competence in vitro [89,90]. Because much of this evidence comes from porcine IVM systems, it remains unclear whether glycine-associated improvements during oocyte maturation are broadly applicable to other livestock species or translate into later reproductive outcomes in vivo. Further comparative studies across livestock species are therefore needed.

### 5.2. Effects of Glycine on Sperm Function and Fertilization Capacity

Spermatozoa are terminally differentiated cells characterized by markedly reduced cytoplasmic volume and loss of most biosynthetic organelles during spermiogenesis, resulting in severely limited endogenous metabolic and transcriptional capacity [91,92]. Such structural constraints severely limit metabolic plasticity [93], rendering spermatozoa highly dependent on the functional integrity of the extracellular microenvironment throughout epididymal transit, ejaculation, and in vitro handling [94,95]. They are also highly vulnerable to environmental perturbations during collection, handling, storage, and assisted reproductive procedures. This vulnerability is particularly evident in spermatozoa of rooster and boar, where membrane lipid composition is enriched in polyunsaturated fatty acids (PUFAs), thereby increasing susceptibility to lipid peroxidation and oxidative injury under in vitro and cryopreservation conditions [96,97,98]. In line with these intrinsic biological constraints, studies investigating glycine in spermatozoa have mainly focused on semen cryopreservation, rather than in vivo male fertility. Most available evidence comes from cattle, where glycine supplementation in freezing extenders has been reported to improve post-thaw sperm motility, membrane integrity, and viability [99]. In other species, including sheep and poultry, the effects of glycine appear to be more variable [100,101], which may reflect species-specific membrane composition, extender systems and cryopreservation protocols rather than a lack of biological activity. These species-dependent responses indicate that glycine cannot yet be considered a uniformly effective additive across male gamete preservation systems. Because mature spermatozoa have limited biosynthetic activity, the available evidence suggests that glycine acts mainly through extracellular mechanisms, including osmotic stabilization, membrane protection, and attenuation of stress-induced cellular damage during cooling, freezing, and thawing [99,100,102]. However, direct evidence that glycine improves fertilization capacity, conception rate, or male reproductive performance in vivo remains limited. Thus, compared with the evidence available for oocyte and embryo culture systems, understanding the role of glycine in male gamete function remains underdeveloped and represents an important direction for future research.

### 5.3. Effects of Glycine on Embryonic Development

Early embryonic development in livestock species such as cattle, sheep, and pigs is characterized by rapid cell division and strong dependence on the extracellular culture environment, particularly in in vitro embryo production (IVP) systems [103,104,105]. The developmental window from cleavage to blastocyst formation is tightly regulated [106], and the physicochemical stability of the culture environment during this period is critical for ensuring normal embryonic progression [107]. In this context, glycine primarily functions to optimize the embryo cryopreservation and culture system supporting developmental progression [108].

In porcine embryo models, supplementation with 6 mM glycine during vitrification and warming significantly increases cleavage and blastocyst formation rates compared with untreated controls [109,110]. These improvements are associated with enhanced cellular stability and better developmental competence after cryopreservation [18,109]. In bovine embryo culture systems, glycine has been incorporated in the form of glycine-glutamine dipeptides as a more stable alternative to free glutamine in modified synthetic oviduct fluid media. Such substitution has been shown to significantly enhance cleavage rates and blastocyst yields in both parthenogenetic and in vitro fertilized embryos, while reducing apoptosis and limiting ammonia accumulation in the culture environment [111,112].

Collectively, these findings suggest that glycine may support early embryonic development under in vitro conditions, possibly by improving aspects of the embryo culture or cryopreservation environment [109,110,111,112]. Because early embryonic development is highly sensitive to metabolic disturbances in the culture environment [113], these in vitro findings should be interpreted with attention to culture conditions and metabolic context. In porcine preimplantation embryos, glycine increased embryo cell number and reduced apoptosis, although these improvements did not lead to live births [18]. Similarly, evidence from bovine embryos indicates that glycine responses may depend partly on osmolarity and culture conditions [19]. Therefore, further studies using implantation, pregnancy, and live-birth outcomes are needed to determine whether the benefits observed in embryo culture systems can be translated into improved reproductive performance in vivo.

### 5.4. Effects of Glycine on Fetal Development

Fetal development in livestock species such as pigs, cattle, and sheep is contingent upon the coordinated regulation of maternal nutrient provision and placental transport efficiency, representing critical determinants of conceptus growth and viability [114]. Gestation is characterized by rapid fetal tissue accretion, which imposes substantial metabolic demands for amino acids (AAs) serving as substrates for protein synthesis, regulators of redox homeostasis, and mediators of cellular proliferation [115].

Under modern production conditions, particularly in hyperprolific sows or during periods of maternal nutrient restriction [116], endogenous glycine biosynthesis capacity is frequently inadequate to meet the augmented metabolic demands of rapid fetal growth [117], thereby rendering glycine conditionally essential during gestation. In porcine models, accumulating evidence indicates that glycine availability plays a critical role in supporting growth and metabolic homeostasis, particularly under conditions of intrauterine growth restriction (IUGR). Dietary glycine supplementation has been shown to improve growth performance and tissue development in IUGR piglets [118], which is associated with enhanced protein synthesis, increased glutathione availability, and reduced oxidative stress in multiple organs [119]. These findings suggest that glycine partially alleviates IUGR-associated impairments through modulation of antioxidant capacity and anabolic metabolism [120].

In ruminants such as sheep and cattle, maternal amino acid availability is also a critical determinant of fetal growth as well [121,122]. Nutrient restriction impairs fetal development, whereas amino acid supplementation can partially restore fetal growth [123,124,125]. Existing studies have reported alterations in amino acid metabolism during gestation [126], but direct evidence for glycine-specific roles in fetal development is still lacking. To facilitate comparison across species, reproductive stages, and experimental systems, the available glycine-related evidence is summarized in Table 1.

## 6. Mechanisms Underlying the Regulatory Effects of Glycine on Reproductive Function

### 6.1. Glycine Regulates Redox Homeostasis and Mitochondrial Function During Oocyte and Early Embryonic Development

Oxidative stress is a major determinant of reproductive failure under both in vitro and in vivo conditions [127]. Elevated levels of ROS compromise mitochondrial integrity, perturb meiotic spindle assembly [128], and trigger apoptosis in oocytes and embryos [129], thereby diminishing their developmental competence [130].

Evidence from in vitro-cultured porcine oocytes has shown that, under metabolic stress conditions, increased ROS accumulation is accompanied by mitochondrial dysfunction and decreased adenosine triphosphate (ATP) production [85], leading to reduced maturation and blastocyst formation rates [131]. Recent studies by the Li group have demonstrated that glycine supplementation in in vitro-cultured porcine oocytes can significantly alleviate adverse effects by restoring redox homeostasis. Specifically, glycine reduces intracellular ROS levels, enhances GSH content, and increases the expression of key antioxidant enzymes, including glutathione peroxidase 4 (*GPX4*), which catalyzes the reduction of lipid peroxides [132]; superoxide dismutase 1/2 (*SOD1/2*), which catalyze the dismutation of superoxide radicals; and catalase (*CAT*), which catalyzes the decomposition of hydrogen peroxide, thereby contributing to the maintenance of intracellular redox homeostasis [21]. These effects attenuate oxidative damage and lipid peroxidation [21]. Furthermore, glycine has been shown to modulate key regulators of redox balance, such as nuclear factor erythroid 2-related factor 2 (NRF2), a master transcription factor controlling antioxidant responses, and solute carrier family 7 member 11 (SLC7A11), a critical component of the cystine/glutamate antiporter system involved in glutathione synthesis, and to suppress ferroptosis under stressful conditions [22]. This modulation provides further support for its role in maintaining intracellular redox homeostasis. Additionally, in cultured porcine oocytes and early embryos, glycine improves mitochondrial membrane potential and increases ATP production, suggesting enhanced mitochondrial function and energy metabolism [110].

Notably, glycine supplementation has been demonstrated to upregulate the expression of pivotal regulators governing mitochondrial homeostasis, such as peroxisome proliferator-activated receptor gamma coactivator 1 alpha (*PPARGC1A*; protein: PGC-1α) and Ras homolog enriched in brain (*RHEB*) [20]. As a master transcriptional coactivator, PGC-1α plays a central role in orchestrating mitochondrial biogenesis and optimizing oxidative phosphorylation efficiency [133,134]. In parallel, RHEB acts as a key upstream activator of mechanistic target of rapamycin complex 1 (mTORC1) signaling, thereby indirectly contributing to the regulation of cellular energy metabolism and mitochondrial function [135]. The elevated transcript levels of these key factors suggest that glycine not only contributes to the maintenance of mitochondrial functional integrity but also facilitates the activation of mitochondrial turnover and adaptive energy metabolic remodeling. Collectively, these effects further enhance the developmental competence of oocytes exposed to stressful in vitro environments.

Similar protective effects have also been demonstrated in embryo models [18]. For example, glycine supplementation has been shown to significantly alleviate oxidative stress in vitrified porcine parthenogenetically activated (PA) 1-cell embryos, as evidenced by reduced intracellular ROS levels, increased GSH content, and the upregulation of antioxidant-related genes such as *SOD2*, *GPX4* and *NFE2L2*. These changes are accompanied by improved mitochondrial membrane potential and ATP production, ultimately leading to enhanced developmental competence and blastocyst quality [109]. Overall, these findings support a potential role for glycine in maintaining redox balance and mitochondrial function under defined in vitro stress conditions. However, most mechanistic evidence comes from porcine models, including ABT-199-induced mitochondrial dysfunction [85], PFOS-induced developmental inhibition [22], and low-glucose culture conditions [20]. Therefore, further studies are needed to determine whether these protective effects are broadly applicable across livestock species and under physiological conditions in vivo.

### 6.2. Glycine Regulates Epigenetic Reprogramming via One-Carbon Metabolism During Early Embryonic Development

Early embryonic development is characterized by extensive epigenetic reprogramming [136], including dynamic changes in DNA methylation and histone modifications [137]. Disruption of these processes is strongly associated with abnormal embryogenesis and impaired developmental potential [138].

In this context, the availability of one-carbon units is a key determinant of cellular methylation capacity, as it supports the synthesis of SAM, which is the universal methyl donor for DNA and histone methylation [139,140]. As an integral component of the folate-mediated one-carbon metabolic network, glycine contributes to the intracellular one-carbon pool and participates in maintaining the metabolic conditions required for efficient methyl group transfer [46]. Consequently, variations in glycine availability may influence cellular methylation capacity during early embryonic development, thereby potentially affecting the fidelity of epigenetic reprogramming [141,142].

Supporting this link between one-carbon metabolism and epigenetic regulation, experimental studies have demonstrated that epigenetic regulation is highly sensitive to cellular metabolic status, particularly the availability of one-carbon substrates required for methylation reactions [143]. In embryonic stem cells, for example, disturbances in one-carbon metabolism reduce the availability of SAM, alter histone methylation patterns (e.g., decreased H3K4me3, a mark associated with transcriptional activation), and hinder cell proliferation [144].

Although direct evidence linking glycine supplementation to epigenetic modifications in animal embryos remains limited, embryonic methylation processes are closely associated with one-carbon metabolism. As a key contributor to one-carbon metabolic pathways [145], glycine may be hypothesized to support epigenetic regulation during early development. However, this proposed relationship remains speculative and requires direct experimental validation in livestock embryos.

### 6.3. Glycine Regulation of AMPK-mTORC1 Pathway in Germ Cell Metabolic Homeostasis and Developmental Competence

Efficient coordination between energy availability and cellular growth is essential for successful oocyte maturation and embryonic development [146,147]. The AMP-activated protein kinase (AMPK)-mechanistic target of rapamycin complex 1 (mTORC1) signaling axis serves as a central regulator of this process by integrating metabolic status with protein synthesis and cell proliferation [148,149,150].

Notably, the regulatory effect of glycine on this signaling pathway appears to be mediated indirectly through improvements in cellular energy status and mitochondrial function. In vitro culture systems with reduced glucose availability have been widely used as a model to mimic metabolic stress in mammalian oocytes and early embryos. Under such conditions, impaired glycolytic flux contributes to reduced ATP production, elevated oxidative stress, and dysregulation of energy-sensing pathways, particularly characterized by increased AMPK activation and suppression of mTORC1 signaling [151], resulting in decreased protein synthesis and compromised oocyte and early embryonic developmental competence [152]. Xu et al. reported that, in an in vitro porcine oocyte model under low-glucose conditions, glycine supplementation partially alleviated metabolic stress by improving mitochondrial function and helping maintain cellular ATP levels. This effect was associated with a partial rebalancing of AMPK-mTORC1 signaling, suggesting a possible role for this pathway in supporting anabolic activity during oocyte maturation and early embryonic development [20]. Increased expression of downstream effectors related to translation initiation and mitochondrial function was also observed, including eukaryotic initiation factor 4E (eIF4E), a key cap-dependent translation initiation factor involved in mRNA translation [149], and PGC-1α, a central regulator of mitochondrial biogenesis and oxidative metabolism [153]. These findings suggest that glycine may improve oocyte maturation and embryonic development under metabolically stressful in vitro conditions.

Beyond this, mTORC1 signaling has also been implicated in the regulation of ovarian follicular dynamics, particularly the activation of primordial follicles [154]. Genetic studies in mouse models have demonstrated that oocyte-specific activation of mTORC1 is sufficient to trigger premature activation of the entire primordial follicle pool, leading to accelerated follicular recruitment and depletion of the ovarian reserve [155]. Conversely, inhibition of mTORC1 activity, such as by rapamycin treatment, preserves follicular quiescence and delays the initiation of follicular growth, indicating that mTORC1 functions as a critical determinant of the transition from follicle dormancy to activation [155,156,157]. These mouse model studies establish a role for mTORC1 in primordial follicle activation, but they do not provide direct evidence that glycine supplementation affects primordial follicle dynamics in vivo. Future studies are therefore needed to directly test whether glycine modulates primordial follicle recruitment in vivo, particularly in livestock models.

**Figure 2:**
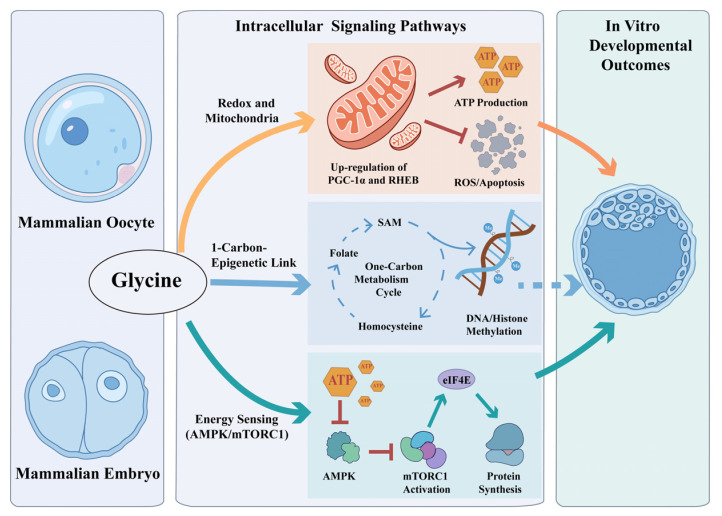
Proposed regulatory pathways linking glycine metabolism to oocyte and embryonic development. This schematic summarizes the potential mechanisms by which glycine metabolism may influence reproductive cell function through redox homeostasis, mitochondrial function, one-carbon metabolism, and AMPK-mTORC1-related energy sensing. Solid arrows indicate pathways supported by glycine supplementation studies in reproductive in vitro models, including redox/mitochondrial regulation and AMPK-mTORC1-related signaling. Dashed arrows indicate indirect or hypothesized links requiring further validation, including the proposed connection between one-carbon metabolism and epigenetic regulation. Red blunt-ended lines indicate inhibition or negative regulation. Abbreviations: PGC-1α, peroxisome proliferator-activated receptor gamma coactivator 1 alpha; RHEB, Ras homolog enriched in brain; eIF4E, eukaryotic initiation factor 4E; AMPK, AMP-activated protein kinase; mTORC1, mechanistic target of rapamycin complex 1; ATP, adenosine triphosphate.

In summary, redox regulation, one-carbon metabolism, and AMPK-mTORC1 signaling may represent interconnected pathways through which glycine metabolism influences reproductive cell function, particularly under in vitro stress conditions. Through effects on mitochondrial activity, methylation-related metabolism, and protein synthesis, these pathways may contribute to oocyte competence and embryonic developmental potential. While substantial progress has been made in characterizing individual mechanisms, the dynamic crosstalk among these pathways, particularly under conditions of metabolic stress, remains poorly defined. Future studies are therefore required to elucidate how glycine-dependent metabolic cues are transduced into coordinated signaling outputs under physiological conditions. A schematic overview of these interconnected regulatory pathways is presented in Figure 2.

## 7. Conclusions and Future Research Directions

Glycine, traditionally classified as a non-essential amino acid, functions as an important metabolic regulator in animal reproduction. Its regulatory roles span multiple levels of cellular homeostasis, primarily through redox homeostasis, one-carbon metabolism, and AMPK-mTORC1 signaling pathways.

### 7.1. Limitations of Current Evidence

Notably, due to the distribution of the available literature, the evidence reviewed here is weighted toward female gametes and embryos and is derived predominantly from in vitro models. Livestock-specific mechanistic data remain limited, and species-dependent differences among gamete, embryo, and fetal models may influence the observed effects of glycine. Moreover, few studies have evaluated long-term reproductive endpoints such as pregnancy rate, litter size, offspring viability, or transgenerational outcomes. These gaps represent a key limitation for translating mechanistic insights into practical livestock production strategies.

### 7.2. Future Research Directions

Future studies should therefore prioritize well-designed in vivo investigations to validate glycine’s reproductive effects under physiological conditions, with particular attention to defining stage-specific requirements, optimizing supplementation strategies, and clarifying interactions with other nutrients and metabolic cofactors. Future work should also examine emerging mechanistic hypotheses, such as the potential crosstalk between glycine metabolism and estrogen receptor-related signaling pathways. Studies in skeletal muscle cells have shown that glycine supplementation can enhance ER-associated signaling and promote downstream anabolic responses [158]. Given the extensive role of estrogen receptors in reproduction, exploring their interaction with glycine in reproductive tissues is meaningful. Ultimately, integrative approaches combining metabolomics, transcriptomics, epigenomic profiling, and functional analyses will be necessary to clarify how glycine-mediated metabolic cues are translated into coordinated reproductive responses and practical nutritional strategies.

## Figures and Tables

**Table 1:** Summary of glycine-related evidence by reproductive stage, species, and experimental system.

Reproductive Stage	Species/Evidence Level	Experimental System	Glycine Concentration/Dose	Key Reported Outcomes	Proposed Process	Species-Specific Insights/Knowledge Gaps
Oocytes	Pig (primary direct evidence); bovine/mouse as contextual evidence	Porcine follicular fluid amino acid analysis; in vitro maturation (IVM) systems	Follicular fluid: 562.01 ± 27.56 μM in small follicles; 336.46 ± 8.82 μM in large follicles; IVM dose varies among studies	Glycine abundance changes during follicular development; supplementation improves oocyte maturation and subsequent embryonic development in porcine IVM systems [21,84]	Osmotic stabilization; redox balance; mitochondrial support; AMPK-mTORC1-related energy sensing [20,88]	Evidence mainly from porcine IVM; translation to in vivo reproductive outcomes remains unverified
Spermatozoa	Cattle (consistent benefits); Sheep, poultry (variable responses)	In vitro semen preservation/cryopreservation	Dose varies by extender and species	Improved post-thaw sperm motility, membrane integrity, and viability in cattle; variable responses in sheep and poultry [99,100,101]	Osmotic stabilization; membrane protection; reduced cryopreservation-induced stress [99,100,102]	Cattle evidence strongest; sheep/poultry variable; fertilization capacity and male fertility in vivo untested
Embryos	Pig, cattle, mouse (direct in vitro evidence)	IVP/IVC; embryo culture; vitrification/warming	Porcine vitrification/warming: 6 mM glycine; bovine media: glycine-glutamine dipeptide forms; dose varies across embryo culture systems	Improved cleavage, blastocyst yield, blastocyst cell number, embryo quality, and reduced apoptosis under culture or cryopreservation conditions [18,109,110,111,112]	Extracellular osmotic stabilization; culture-environment stability; reduced apoptosis; possible mitochondrial support [108,113].	Evidence confined to culture/cryopreservation models; implantation, pregnancy, live birth, and offspring outcomes untested
Fetus	Pig IUGR-related models	In vivo dietary supplementation	0.2–0.8 g/kg BW/day orally; 0.4 g/kg BW/day most cost-effective	Improved growth of sow-reared IUGR piglets and enhanced tissue glutathione availability in IUGR pigs [119,120]	Glutathione synthesis support; antioxidant defense; protein synthesis and growth-related metabolism [119,120]	Physiologically relevant but incomplete; direct evidence in ruminant fetal development remains lacking

Abbreviations: IVM, in vitro maturation; IVP, in vitro production; IVC, in vitro culture; IUGR, intrauterine growth restriction; BW, body weight. Primary direct evidence indicates studies directly testing glycine or glycine-containing supplementation in the indicated reproductive model; contextual evidence indicates supporting background from related species, models, or mechanisms.

## Data Availability

No new data were created or analyzed in this study. Data sharing is not applicable to this article.

## Abbreviations

AMPK, AMP-activated protein kinase; AKT, Protein kinase B; ATP, Adenosine triphosphate; CAT, Catalase; DLD, Dihydrolipoamide dehydrogenase; eIF4E, Eukaryotic initiation factor 4E; ERα, Estrogen receptor alpha; ERβ, Estrogen receptor beta; GCS, Glycine cleavage system; GCSH, Glycine cleavage system protein H; GLDC, Glycine decarboxylase; GNMT, Glycine N-methyltransferase; GPX4, Glutathione peroxidase 4; GSH, Glutathione; IUGR, Intrauterine growth restriction; IVM, In Vitro Maturation; mTOR, Mechanistic target of rapamycin; mTORC1, Mechanistic target of rapamycin complex 1; NADH, Nicotinamide adenine dinucleotide (reduced form); NRF2, Nuclear factor erythroid 2-related factor 2; PI3K, Phosphoinositide 3-kinase; PGC-1α, Peroxisome proliferator-activated receptor gamma coactivator 1 alpha; RHEB, Ras homolog enriched in brain; ROS, Reactive oxygen species; SAM, S-adenosylmethionine; SHMT, Serine hydroxymethyltransferase; SHMT1, Cytosolic serine hydroxymethyltransferase 1; SHMT2, Mitochondrial serine hydroxymethyltransferase 2; SLC7A11, Solute carrier family 7 member 11; SOD1, Superoxide dismutase 1; SOD2, Superoxide dismutase 2; THF, Tetrahydrofolate; 5,10-CH_2_-THF, 5,10-methylenetetrahydrofolate; 5-CH_3_-THF, 5-methyltetrahydrofolate.
